# Loss of *Usp9x* Disrupts Cortical Architecture, Hippocampal Development and TGFβ-Mediated Axonogenesis

**DOI:** 10.1371/journal.pone.0068287

**Published:** 2013-07-05

**Authors:** Shane Stegeman, Lachlan A. Jolly, Susitha Premarathne, Jozef Gecz, Linda J. Richards, Alan Mackay-Sim, Stephen A. Wood

**Affiliations:** 1 Eskitis Institute for Cell and Molecular Therapies, Griffith University, Brisbane, Queensland, Australia; 2 Neurogenetics Research Program, South Australian Pathology at Women’s and Children’s Hospital, Adelaide, South Australia, Australia; 3 School of Paediatrics and Reproductive Health, The University of Adelaide, Adelaide, South Australia, Australia; 4 Queensland Brain Institute and The School of Biomedical Sciences, The University of Queensland, Brisbane, Queensland, Australia; Universitat Pompeu Fabra, Spain

## Abstract

The deubiquitylating enzyme Usp9x is highly expressed in the developing mouse brain, and increased Usp9x expression enhances the self-renewal of neural progenitors *in vitro*. *USP9X* is a candidate gene for human neurodevelopmental disorders, including lissencephaly, epilepsy and X-linked intellectual disability. To determine if *Usp9x* is critical to mammalian brain development we conditionally deleted the gene from neural progenitors, and their subsequent progeny. Mating *Usp9x^loxP/loxP^* mice with mice expressing *Cre* recombinase from the Nestin promoter deleted Usp9x throughout the entire brain, and resulted in early postnatal lethality. Although the overall brain architecture was intact, loss of Usp9x disrupted the cellular organization of the ventricular and sub-ventricular zones, and cortical plate. Usp9x absence also led to dramatic reductions in axonal length, *in vivo* and *in vitro*, which could in part be explained by a failure in Tgf-β signaling. Deletion of Usp9x from the dorsal telencephalon only, by mating with *Emx1-cre* mice, was compatible with survival to adulthood but resulted in reduction or loss of the corpus callosum, a dramatic decrease in hippocampal size, and disorganization of the hippocampal CA3 region. This latter phenotypic aspect resembled that observed in Doublecortin knock-out mice, which is an Usp9x interacting protein. This study establishes that Usp9x is critical for several aspects of CNS development, and suggests that its regulation of Tgf-β signaling extends to neurons.

## Introduction

During embryonic development of the brain, neural cells need to respond rapidly to changing environmental cues. In the developing axon and dendrites, these decisions are made at a distance from the nucleus and so rely heavily on post-translational mechanisms. The ubiquitin system regulates protein stability, localisation and function in a rapid and quantitative manner and has been shown to regulate multiple aspects of neural development [1] [2] [3] [4]. Not surprisingly, given the precipitous consequences of protein ubiquitylation, defects in the ubiquitin system have been linked to a range of neurodevelopmental and neurodegenerative diseases [5] [6] [7] [8] [9]. Specificity in the ubiquitin system is imparted by the hundreds of E3 ubiquitin ligases and deubiquitylating enzymes (DUBs), which add or remove ubiquitin, respectively. DUBs function downstream in the ubiquitin pathway, thus having the potential to act as final arbiters of protein substrate fate and function [10] [11] [12]. Several studies have shown that DUBs play important roles in the growth, function and maintenance of neurons and synapses [13] [14], [15].

Ubiquitin specific protease 9, located in the X chromosome (*Usp9x*, also called FAM), is a substrate-specific DUB that is highly expressed in the developing CNS in both humans and mice [16]–[19]. Although *Usp9x* expression decreases in the mature CNS it remains strongly expressed in the neurogenic regions including, the sub-ventricular zone of the lateral ventricles and the sub-granular zone cells of the dentate gyrus [17], [20]. *Usp9x* function has been implicated in several aspects of CNS development. Increased expression of *Usp9x* in embryonic stem cell-derived neural progenitors promotes their organisation into polarised clusters, and increases their self-renewal and cellular potency [17]. In Drosophila, *Usp9x*’s homologue, *fat facets (faf)* regulates photoreceptor fate as well as synaptic morphology and function [13], [21]. In humans *USP9X* has been implicated in lissencephaly and epilepsy [16] and is an X-linked Intellectual Disability candidate gene [22].

Usp9x is a large DUB (2554 amino acids) and several of its substrates regulate aspects of neural development and/or homeostasis. These include components of neurodevelopmental signalling pathways such as Notch [23]–[25], Wnt [26] and TGF-β [27]. In the Notch pathway Usp9x regulates the trafficking accessory protein Epsin [28], as well as the ubiquitin ligase Mind Bomb1 [29] [30] in the signal sending cells. Usp9x also stabilises the Notch intracellular domain E3 ligase, Itch [31], which functions in the signal receiving cell [23], [25]. Usp9x directly binds and stabilises β-catenin, a component of cell-cell adhesion and a Wnt signalling pathway second messenger, in a range of the mammalian cells and tissues, including the CNS [26], [32], [33] where it is required for proper development [34]–[36]. Usp9x deubiquitylation of Smad4 is essential for signalling by members of the Tgf-β family [27], [37].

Still other Usp9x substrates regulate neural progenitor adhesion and proliferation. Acute lymphoblastic leukemia-1 fusion partner from chromosome 6 (AF-6) is essential for the establishment of adherens junctions and polarity in neural progenitor cells [38], [39]. Usp9x regulates both the stability and localisation of AF-6 [40] [41]. Another Usp9x substrate, Activator of G protein Signalling 3 (AGS3) is involved in spindle orientation and asymmetric cell division in cortical progenitors [42] [43]. Finally, Usp9x binds the microtubule-associated protein Doublecortin (DCX), which is involved in neuronal migration, protein sorting and vesicle trafficking [16] [44]. The interaction between Usp9x and DCX is clearly important for human CNS development as patients with point mutations in DCX that cannot bind Usp9x, develop lissencephaly [16].

The above evidence suggests that there are ample avenues through which Usp9x might regulate CNS development. However, there are two significant caveats to implying a role for Usp9x in neural development *in vivo* based simply on the observed substrate associations. These are, (i) most of the Usp9x-substrate interactions and regulation have been determined in cultured cells; (ii) whether or not Usp9x is the rate-limiting determinant of a substrate’s fate, and any subsequent developmental consequences, is largely cell context-specific. Therefore, to assess the requirement for Usp9x during mammalian CNS development we conditionally deleted the *Usp9x* gene in neural progenitors using tissue-specific *Cre recombinase* gene expression. Loss of Usp9x in all neural progenitors resulted in early post-natal lethality, and was associated with disorganised cortical architecture and reduced corpus callosum and hippocampal volumes. More detailed analysis of neurons revealed neurite growth defects that can, in-part, be explained by refractory responses to TGFβ stimulation. The results demonstrate novel roles for *Usp9x* in brain development.

## Materials and Methods

### Ethics Statement

All experiments were performed under ethical clearance from the Griffith University, and The Women’s and Children’s Health Network, and the South Australian Pathology Department Animal Ethics Committees. The research was conducted in accordance with the policy and guidelines of the National Health and Medical Research Council of Australia. Animals were monitored for signs of pain and distress, all euthanasia was performed using cervical dislocation, and all efforts were made to minimize suffering.

### Generation of *Usp9x^loxP^* Mice

*Usp9x^loxP^* mice were generated by Ozgene *Pty Ltd*, Bentley, Australia, as described [45]. Briefly, *loxP* sites were incorporated into the second and third introns flanking exon three. Initiation of translation occurs in exon two, followed by 96bps of coding sequence. Deletion of exon three (146 bp), which contains an incomplete number of codons, would result in a frame shift with the next six alternative ATG start codons out of frame. To assess if any translation occurred from an in frame start codon downstream of exon three, an antibody raised against the USP9X C-terminal was used.

### Deletion of *Usp9x* in the Developing Mouse Brain

*Usp9x^loxP/loxP^* female mice were crossed with *Nestin-Cre* [46] or *Emx1-Cre* [47] males, to delete *Usp9x* from the whole brain (Nestin-cre) or dorsal telencephalon (Emx1-cre), respectively. Using this breeding scheme *Usp9x* would be deleted from males, which inherited *Cre recombinase* and in this gender result in a *Usp9x* null genotype. *Cre*-negative male littermates were used as negative controls in all experiments, except where noted. *Cre*-positive female offspring are heterozygous for *Usp9x* gene deletion.

For all analyses a minimum of three or more *Usp9x^cKO^/Y* mice versus three or more littermate controls were used. Results were assessed statistically using a Student’s *t* test unless otherwise specified.

### Mouse Genotyping

DNA was extracted from neural tissues (brain or spinal cords) and PCR was performed using standard techniques. Primers were designed to detect *Cre*-*recombinase*: for 5′-TGATGAGGTTCGCAAGAACC, rev 5′-CCATGAGTGAACGAACCTGG. Male embryos were identified using primers for the *Sry* region of the Y chromosome: for 5′-GAGGCACAAGTTGGCCCAGCAG, rev 5′-GGTTCCTGTCCCACTGCAGAAG. *Usp9x* primers: for 5′-GCTCACCATTAGGTTGTTAG, rev 5′-TAGACCCATCATGAACCATG. *Usp9x* primers detect wild type *Usp9x* (505 base pairs) as well as the *Usp9x^loxP^* gene with exon three removed → *Usp9x^cKO^/Y* (207 base pairs).

### Reverse Transcription-PCR Analysis

Total RNA was extracted from brains or testes using TRIzol reagent (Invitrogen). Total RNA was treated with DNase I (Invitrogen) then subjected to a mRNA purification step using a MicroPoly(A) Purist mRNA purification kit (Ambion). Reverse Transcription was performed using SuperScript III Reverse Transcriptase primed with oligo(dT) (Invitrogen). PCR was then performed on cDNA using standard techniques. *Usp9y* primers: F 5′-ATGGCAGGTTGCACATTCAC, R5’-GTCTTCATTACCCTGCAAGATC. qPCR reactions on RNA isolated from hippocampal neurons were generated using the iTaq SYBR Green Supermix (Biorad), run on the StepOne Plus Real Time PCR System and analysed using StepOne Sortware V2.0 (Applied Biosystems). Primers include; *Bdnf* F: ACTGGCTGACACTTTTGAGC, R: GCGTCCTTATGGTTTTCTTCG; *Hes1* F: AATGACTGTGAAGCACCTCC R: GTTCATGCACTCGCTGAAGC; *EphB2* F: GTTGTATCTCAGATGATGATGG R: GTCAAACCTCTACAGACTGG; *Runx1* F: TCTGCAGAACTTTCCAGTCG R: GAGATGGACGGCAGAGTAGG.

### Western Blot Analysis of Brain Tissue

Protein was extracted from embryonic and adult brain tissues and Western blots were performed as described previously [33]. Signal was detected using horseradish peroxidase-conjugated secondary antibodies (Millipore) developed with Immobilon Western Chemiluminescent HRP Substrate (Millipore) then imaged on a VersaDoc 4000 MP Imaging System (BioRad).

### Histology and Immuno-fluorescence on Brain Sections

For analysis of embryonic brains, samples were drop fixed in 4% paraformaldehyde. For adult brains animals were anesthetized, perfused trans-cardially with 4% PFA then heads were drop-fixed in 4% PFA. Following fixation brains were processed for paraffin or cryo-sectioning using standard techniques and sectioned at 10 µm. Histological analyses were performed using standard cresyl violet (Nissl) staining. Hippocampal area was calculated using SPOT software (Diagnostic Instruments Inc). The hippocampal area of the first control was designated 100. The hippocampal areas of all other controls and *Usp9x^cKO^/Y* mice were converted to ratios compared to the first control. For immuno-fluorescence brain sections were blocked with normal donkey serum (Invitrogen), incubated overnight at 4°C with primary antibodies, then incubated for 3 h at room temperature with secondary antibodies and mounted with Vectashield mounting medium with DAPI (Vector Laboratories). Images were obtained on a AxioImager Z1 microscope (Carl Zeiss) or Olympus FV1000 confocal microscope. For coronal analysis, sections from a comparable position along the rostral-caudal axis were used [48]. Sections were matched by counting the number of coronal sections starting at the rostral-most edge of the brain and confirmed by closely matching any unchanged anatomical land marks [49].

### Neuronal Cell Assays

For hippocampal-derived neuronal cultures, embryos were harvested at E18.5 and hippocampal neurons isolated and cultured as previously described [50]. For morphometric analysis, isolated cells were transfected with pMAX-*EGFP* using the mouse neuron nuclefector kit according to the manufactures instruction (Lonza). Cells were fixed with 4% PFA and immuno-fluorescence was performed as previously described [50]. Images were generated on an Axioplan2 microscope (Carl Zeiss). Axons and dendrites were identified using Tau1 and MAP2 immuno-reactivity respectively, and measured using ImageJ software (National Institute of Health). Only neurites10 µm in length or longer were included. For neurite growth kinetic analysis *Usp9x^cKO^/Y* cultures from five different embryos were compared with littermate control cultures from four different embryos. At least twenty neurons were analysed per culture. For neurite growth in response to TGFβ signalling, day 3 cultures were supplemented with 1 ng/ml TGFβ (eBioscience) and cultured for 48 hours before fixation and analysis. Cultures were derived from 3 different *Usp9x^cKO/Y^* embryos and 3 different littermate control embryos. At least 20 neurons were scored in each culture. For transcriptional response to TGFβ signalling, day 3 pooled cultures derived from 3 *Usp9x^cKO/Y^* embryos and 3 littermate embryos were treated with 1 ng/ml TGFβ for 48 hours and RNA analysed by qRT-PCR as described above. Technical triplicate reverse transcriptase reactions were analysed. For TGFβ luciferase reporter assays, isolated neurons were co-transfected with 5 ug of the SMAD3/4 reporter construct pGL3-CAGA-Luc [51]; Kind gift of Dr Hong-Jian Zhu, University of Melbourne) and 50 ng pGL4.74-*Renilla* (Promega) using nucleofection as previously described. This experiment was conducted on cultures isolated from 5 *Usp9x^cKO/Y^* embryos and 5 control littermates grown in duplicate. Following 2 days of culture, 0–10 ng/ml TGFβ was added to the media for 24 hours. Each culture was lysed and analysed in technical triplicate using the Dual Luciferase Reporter Assay System as per manufactures instructions (Promega). Control experiments were conducted using un-transfected cells, and cells transfected with *Renilla* or *Luciferase* only. All graphs display mean average of replicate experiments, error bars represent standard deviations, and statistically analysed using students unpaired 2-tailed t-test, a p-value of less than or equal to 0.05 was considered significant.

### Primary Antibodies

Rabbit (Rb) anti-USP9X (Bethyl Laboratories A301-351A), Rb anti-GapDH (R&D Systems 2275-PC-100), Rb anti-NF160 (Abcam ab9034), Chicken (Ck) anti-MAP2 (Millipore AB15452), Mouse (Ms) anti-Tau1 (Millipore AB1512), Ms anti-MAP2 (Sigma-Aldrich M 1406), Rb anti-GFAP (DakoCytomation Z0334), Ck anti-MAP2 (Chemicon), Ms anti-04 (Millipore MAB345), Rb anti-Cleaved caspase-3 (Cell Signaling Technology 9661), Rb anti-DCX (Abcam ab18723), Rabbit anti-BLBP (Millipore ABN14).

### Secondary Antibodies

Dk anti-rabbit Alexa-Fluor 594 (Invitrogen), Dk anti-mouse Alexa-Fluor 647 (Invitrogen), Dk anti-chicken Cy3 (Jackson Laboratories), Dk anti-mouse Alexa-Fluor 488 (Invitrogen), Gt anti-rabbit HRP (Millipore).

## Results

### Deletion of *Usp9x* from the Developing CNS Results in Perinatal Lethality

As *Usp9x* is required for pre-implantation mouse embryo development [41], in order to study *Usp9x’s* role in brain development we generated *Usp9x^loxP/loxP^* females and bred them with heterozygous males expressing *Cre* recombinase from the Nestin promoter-enhancer, which is active in all CNS neural progenitors from E10.5 [52]. Using this strategy, males that inherited the *Nestin*-*Cre* transgene, would be potentially *Usp9x* null (hereafter referred to as *Nes*-*Usp9x^−/Y^* mice). In preliminary studies using mouse ES cells *in vitro*, we had established that activation of *Cre* resulted in the loss of *Usp9x* exon3 and the Usp9x protein (data not shown). The efficiency of *Usp9x* exon3 and protein deletion in the brain *in vivo* was initially evaluated in E18.5 *Usp9x^−/Y^* embryos (Figure S1A,B) by PCR and immunoblot using a C-terminally targeted Usp9x antibody (Figure S1B). Immunoblots using an antibody against the N-terminal 20 amino acids of Usp9x showed a similar reduction in full-length (290kDa) Usp9x levels (data not shown). We observed some residual full-length Usp9x protein, but this was likely due to blood vessels and brain meninges that were present in the samples [53] [54] [52]. Immunofluorescence analyses detected very low, residual levels of Usp9x protein in neural cells in E12.5 embryos (n = 4) and confirmed complete loss of Usp9x protein in *Nes*-*Usp9x^−/Y^* embryos by E14.5 (n = 4 embryos) (Figure S.1 C–F).

*Nestin-Cre* mediated loss of Usp9x throughout the brain resulted in early postnatal lethality. Of 154 pups analyzed, from 18 litters, all *Nes*-*Usp9x^−/Y^* male mice died within 24 hours of birth. Although *Nes*-*Usp9x^−/Y^* males could move at birth, were pink and appeared to breathe normally, they failed to suckle, as evidenced by lack of milk in their stomachs at the time of death. Female offspring, heterozygous for the knockout of *Nes*-*Usp9x* (*Usp9x^−/X^*), appeared normal at birth and survived to adulthood at rates similar to wild-type females.

*Usp9x* has a homologue on the Y chromosome, *Usp9y*. Although *Usp9y* is reported to only be expressed during spermatogenesis in the mouse [55], we performed RT-PCR on P0 mouse brains to see if its expression is induced following the deletion of *Usp9x*. We failed to detect *Usp9y* expression in these brains indicating that it is not induced and potentially compensating for some *Usp9x* functions (Figure S2).

### Loss of Usp9x Results in Reduced Neuronal Processes

**Figure 1:**
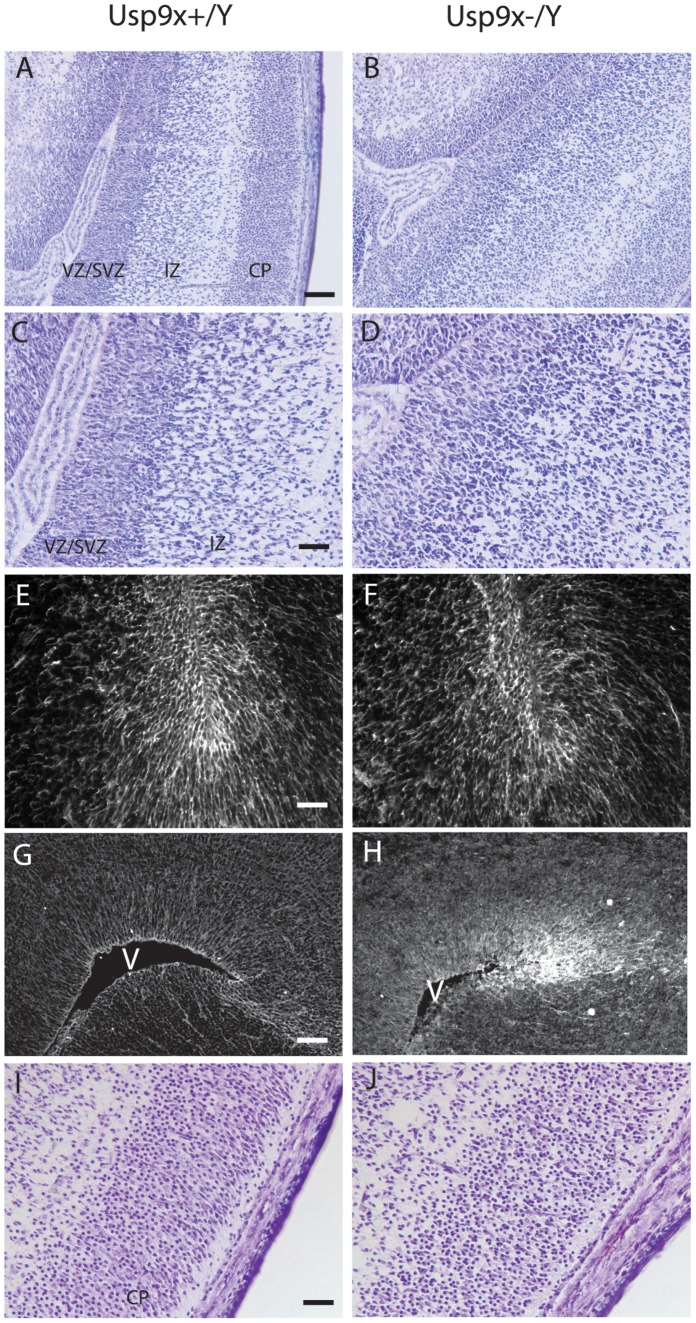
Loss of *Usp9x* disrupts the architecture of the embryonic neocortex. The Nestin-cre mediated deletion of Usp9x (B,D) results in loss of demarcation between the cells of the ventricular and sub-ventricular zones (VZ/SVZ), the more disperse cellular density of the intermediate zone (IZ) and the neurons of the cortical plate (CP) seen in control littermates (A,C). C and D are higher magnification images of A and B, respectively. Nestin (E,F) and BLBP (G,H) staining in E18.5 embryos indicated that neural progenitors were more loosely organized in the VZ/SVZ. Neurons of the cortical plate were disorganized in the absence of *Usp9x* (J) compared with littermates (I). Nissl stain of *Usp9x^+/Y^* (A,C,G) and *Usp9x^−/Y^* (B,D,H) in E16.5 embryos (A–D, G–H). V = ventricle. Scale bar = 100 µm (A), 50 µm (C), 40 µm (E), 100 µm (G), 40 µm (I).

Analysis of E16.5 to E18.5 brains failed to detect a significant decrease in the size of *Nes*-*Usp9x^−/Y^* brains and low power histological analysis revealed that all major CNS regions were present in the absence of Usp9x (not shown). However Nissl staining revealed a general disorganization of the brain architecture. The most obvious was the loss of a clear demarcation between the neural progenitors in the ventricular and subventricular zones and the neuroblasts of the intermediate zone in E16.5 embryos (Fig. 1A–D) and E17.5 (not shown). These observations were confirmed in E18.5 embryos where the neural progenitor and radial glial markers Nestin and Brain Lipid Binding Protein (BLBP) were more diffusely localized and disorganized in *Usp9x^−/Y^* embryos (Fig. 1E,H). The neurons of the cortical plate were also less densely packed in the absence of Usp9x (Fig. 1I,J).

**Figure 2:**
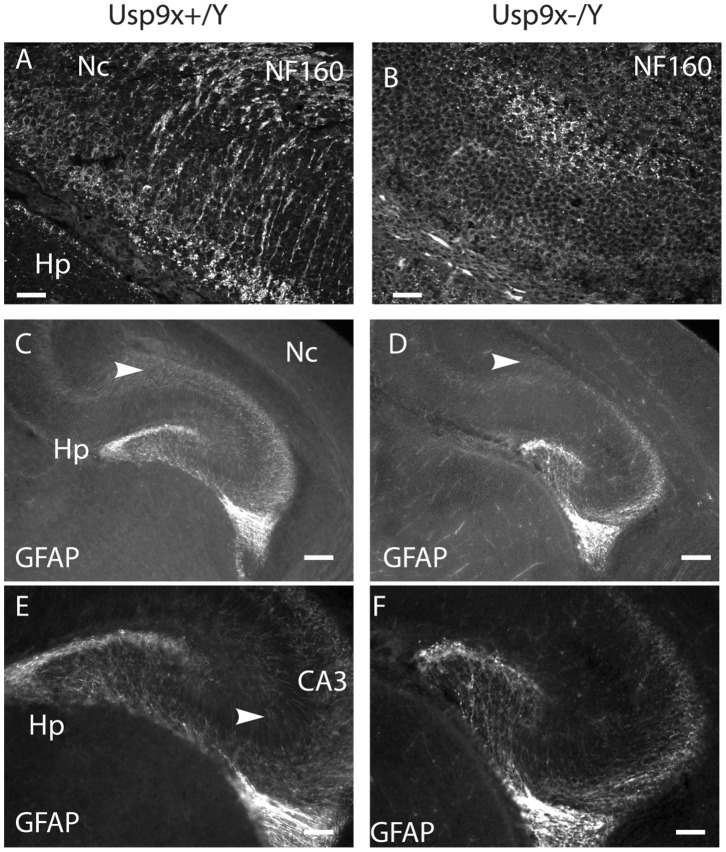
*Usp9x* loss affects neuronal and astrocytic projections. NF160 antibodies decorate axonal projection from the neocortex (Nc) to the hippocampus (Hp) in E18.5 Nes-*Usp9x^+/Y^* mice (A). These projections were absent in Nes-*Usp9x^−/Y^* mice (B). GFAP staining is reduced in both the hippocampus and neocortex of E18.5 *Usp9x ^−/Y^* embryos (D) compared with littermate controls (C). In the hippocampus GFAP-labeled projections extended toward the CA3 region in control embryos (arrowhead in E) but not in the absence of *Usp9x* (F). Scale bar = 20 µm (A,B), 160×µm (C,D), 80×µm (E,F).

We next sought to determine the affect of Usp9× loss on neurons and glia by staining for NF160 and GFAP, respectively. In the absence of Usp9x we detected a dramatic reduction in the number and length of NF160-positive neuronal processes. This was most evident in those projecting from the entorhinal cortex towards the hippocampus (Fig. 2A,B). GFAP staining was reduced in size in the hippocampus and did not extend as far medially in the cerebral cortex in E18.5 embryos (Fig. 2C,D). Within the dentate gyrus of the hippocampus GFAP stained projections were not as extensive in the absence of Usp9x (Fig. 2C–F).

As *Nes*-*Usp9x^−/Y^* mice die at P0, to study the effects of *Usp9x* depletion on post-natal stages of brain development we mated *Usp9x^loxP/loxP^* females to heterozygous *Emx1*-*Cre* males, where *Cre* expression is restricted to the neural progenitors of the dorsal telencephalon from E9.5 onwards [56]. Loss of almost all the dorsal telencephalon is compatible with survival [57] and indeed *Emx1*-*Cre* mediated *Usp9x* knockout mice (referred to as *Emx1*-*Usp9x^−/Y^* hereafter) survived into adulthood. In an attempt to generate *Emx1-Usp9x^−/−^* females we paired four adult *Emx1-Usp9x^−/Y^* males with eight *Usp9x^loxP/loxP^* females over a period spanning four months, however, no litters were produced. Therefore, *Emx1- Usp9x^−/Y^* mice fail to produce viable offspring.

**Figure 3:**
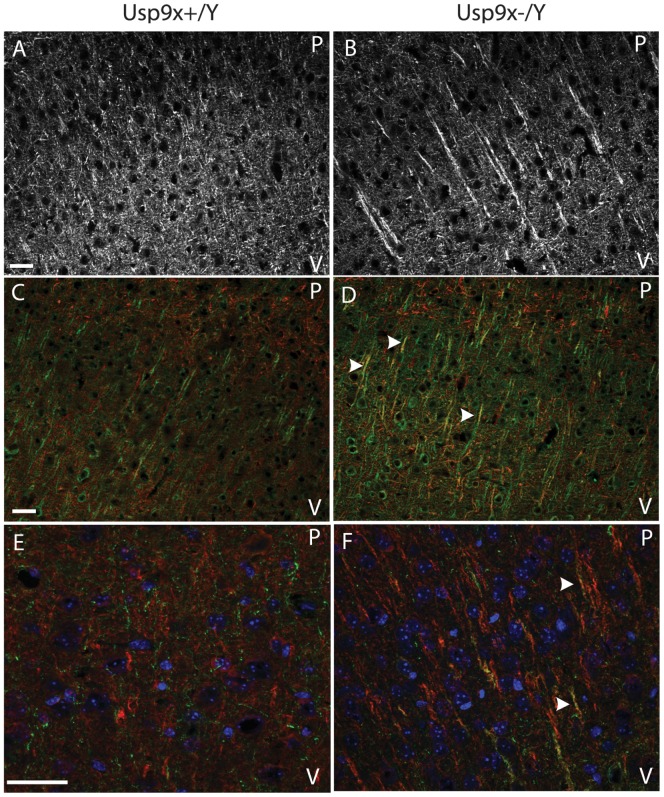
Absence of *Usp9x* alters neuronal projections in the neocortex. NF160 staining of adult cerebral cortex (7–8 wk) Emx1-*Usp9x^+/Y^* (A,C,E) and Emx1-*Usp9x^−/Y^* (B,D,F). NF160 stains thick, pial-oriented projections in Emx1-*Usp9x^−/Y^* mice (B). NF160 (red) colocalises with the dendritic marker MAP2 (green) in the thick projections detected in Emx1-*Usp9x^−/Y^* cortical neurons (arrowheads in D,F). There is little overlap in control littermates (C,E). Orientation is such that the pial surface (P) is to the top, and ventricle (V) to the bottom of each image. Scale bar = 30 µm (A), 40×µm (C,E).

In *Emx1*-*Usp9x^−/Y^* mice, *Emx1*-*Cre* activity resulted in loss of Usp9x protein from the telencephalon (Figure S5). As observed in *Nes*-*Usp9x^−/Y^* embryos, the neocortex of *Emx1*-*Usp9x^−/Y^* mice had reduced neuronal processes projecting through the entorhinal cortex towards the hippocampus (not shown). In adult (7–8 wk old) *Emx1*-*Usp9x^−/Y^* mice NF160 immunoreactivity detected aberrant neuronal processes (Fig. 3 A,B). In the absence of Usp9x, NF160-immunoreactive processes were much thicker and predominantly projected toward the pial surface (top of image), rather than ventricle (Fig. 3A,B). Aberrant processes were found in all cortical layers. The localisation of the dendritic marker MAP2 was similar in the presence or absence of Usp9x. However while the MAP2 and NF160 stains did not overlap in control littermates they partially co-localised in the thick pial-orientated projections in *Emx1*-*Usp9x^−/Y^* brains (Fig. 3C–F).

**Figure 4:**
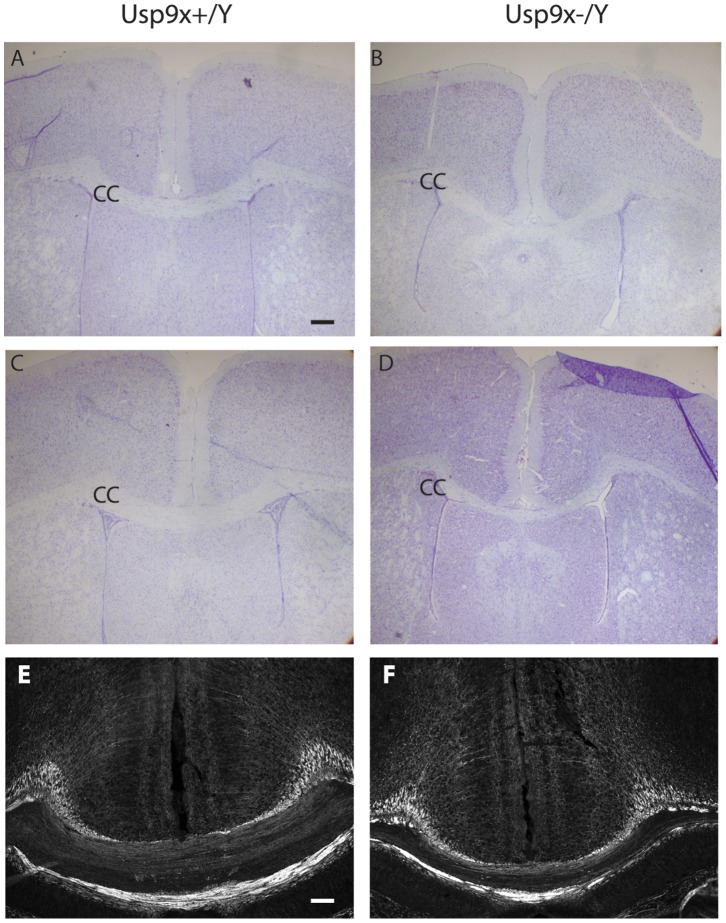
Loss of *Usp9x* results in reduction of the corpus callosum. Nissl staining of matched sections showing thinning of the corpus callosum In Emx1-*Usp9x^−/Y^* adults males (C,D) compared with Emx1-*Usp9x^+/Y^* littermates (A,B). NF160 staining revealing reduction of the corpus callosum is evident in Emx1-*Usp9x^−/Y^* P8 mice (E,F). (n = 4 for both Emx1-*Usp9x^−/Y^* and Emx1-*Usp9x^+/Y^* littermates). Scale bar = 160 µm (A), 200 µm (E).

The corpus callosum is the major axonal tract connecting the two cerebral hemispheres. Analysis of the corpus callosum, from its most rostral to caudal aspects, revealed a reduction in the dorso-ventral thickness in *Emx1*-*Usp9x^−/Y^* mice (Fig. 4A–F). These data suggest that Usp9x is required for the growth of axon tracts in the brain.

Usp9x binds Doublecortin (Dcx) protein which is important for neuronal migration in the human cortex [58]. Loss of Usp9x however, in both *Nes-Usp9x^−/Y^* (E12.5– E18.5) and *Emx1-Usp9x^−/Y^* (P7) mice did not affect the overall localization or level of Dcx in the developing cerebral cortex (Figure S3).

### Deletion of Usp9x Results in Reduced Hippocampal Size

**Figure 5:**
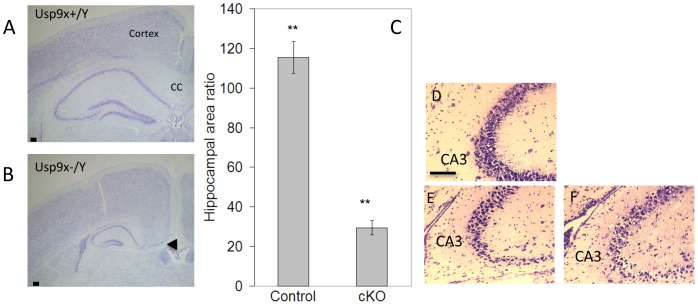
*Usp9x* is required for hippocampal development. The hippocampi of adult Emx1-*Usp9x^−/Y^* mice (B) were reduced in area compared with *Usp9x^+/Y^* littermates (A). Nissl stain of 7–8week old mice. (C) Quantification of hippocampal area of *Usp9x^+/Y^* adult males (Control, n = 4) compared with *Usp9x^−/Y^* (cKO, n = 4) (** p<0.01). (D–F) Higher magnification identifying disruption and reduction of the CA3 region in *Usp9x ^−/Y^* males. (D - *Usp9x^+/Y^*; E,F independent *Usp9x^−/Y^* males) Scale bar = 100 µm (A,B), 150 µm (D–F).

Loss of Usp9x in the forebrain also dramatically affected the size of the hippocampus. Analysis of hippocampal area from matched sections detected a 74% reduction in hippocampal area in adult *Emx1-Usp9x^−/Y^* mice (Fig. 5A–C). A similar degree of reduction in the absence of Usp9x was evident from the most rostral to caudal aspects of the hippocampus. We also analysed the hippocampi of adult *Emx1-Usp9x^−/X^* heterozygous females and detected a reduction of hippocampal area, though to a lesser extent (data not shown).

Despite the reduction in size, most regions of the adult hippocampus retained their relative organisation and cytoarchitecture. However the cytoarchitecture of the CA3 region of the stratum pyramidale was particularly affected (Fig. 5 D–F). There was a significant thinning of the CA3 region in *Emx1-Usp9x^−/Y^* mice (48.51+/−0.21 µm SEM (*Usp9x^+/Y^*, n = 6); 21.95+/−6.43 µm SEM (*Emx1-Usp9x^−/Y^*, n = 5, p = 0.011)). There were no significant alterations in the thickness of CA1 or CA2 areas, or within the granular cell layers of the dentate gyrus in Emx1-*Usp9x^−/Y^* mice (data not shown). A less severe reduction in hippocampal size was already apparent in E17.5 and E18.5 embryos (data not shown).

A reduction in cell number may result from decreased proliferation, increased apoptosis or a reduction in the migration of neurons or glia within the hippocampus during development. Analysis of apoptosis by staining for cleaved caspase-3 [48] (Figure S4.) revealed that loss of Usp9x increased apoptosis in the hippocampus and medial neocortex at E18.5 and P0 (*Usp9x^+/Y^* = 1.1+/−1.1 cells (n = 9) versus *Usp9x^−/Y^* 5.9+/−5.1 cells, n = 10; p = 0.013).

### Deletion of Usp9x Results in Reduced Axonal Elongation and Tgf-β Response *in vitro*

**Figure 6:**
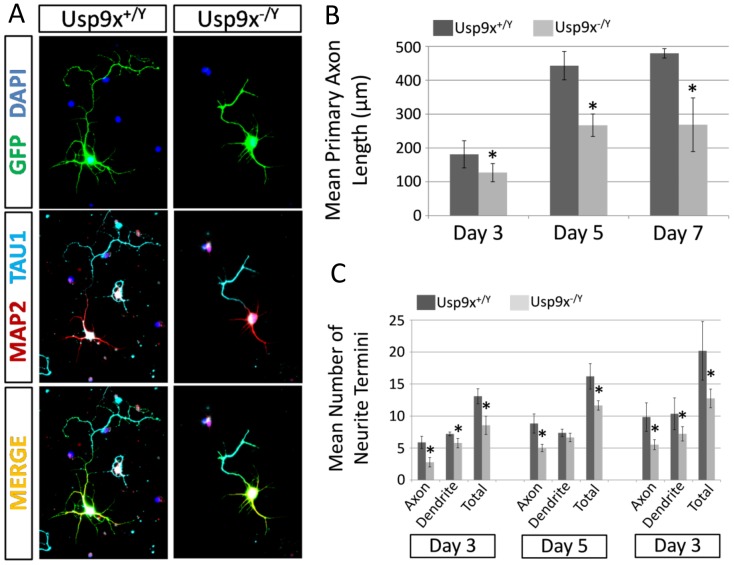
Loss of *Usp9x* reduces neuronal outgrowth. Embryonic hippocampal neurons were isolated, transfected with a plasmid encoding Enhanced Green Fluorescent Protein, and grown in-vitro for 3, 5 or 7 days. (a) Example immunofluorescent images of wildtype (Nes-*Usp9x^+/Y^*) and null (Nes-*Usp9x^−/Y^*) neurons resolved using GFP expression (Green) and co-stained with the axonal and dendritic specific antibodies, TAU1 (cyan) and MAP2 (red) respectively. (b–c) Morphometric analysis was employed to record mean primary axonal length (b) and number of neurite termini (c). *p<0.05 by student 2-tailed t-test.

To determine if reduced axon length was due to cell autonomous effects of Usp9x, we cultured hippocampal neurons *in vitro* and measured both neurite length and branching. [59]. Cultured Nes-*Usp9x^−/Y^* hippocampal-derived neurons displayed both reductions in primary axonal length and in the number of axonal and dendritic termini, which reflects their degree of arborisation (Fig. 6). At day 3 of culture, *Usp9x^−/Y^* axons were 30% shorter than control axons, whilst by day 7 of culture this difference had reached 44% (Fig. 6B). Likewise, the total number of neurite termini was reduced by 28–36% across all days of culture, and whilst the reduction in axonal termini number was the predominant contributor to this result (with reductions ranging between 43–53%), reductions in dendritic termini were also observed (Fig. 6C). Given the low cell density in these experiments the data strongly suggest that Usp9x function is required cell autonomously for the initiation and/or elongation of neurites, which supports our *in vivo* observation.

**Figure 7:**
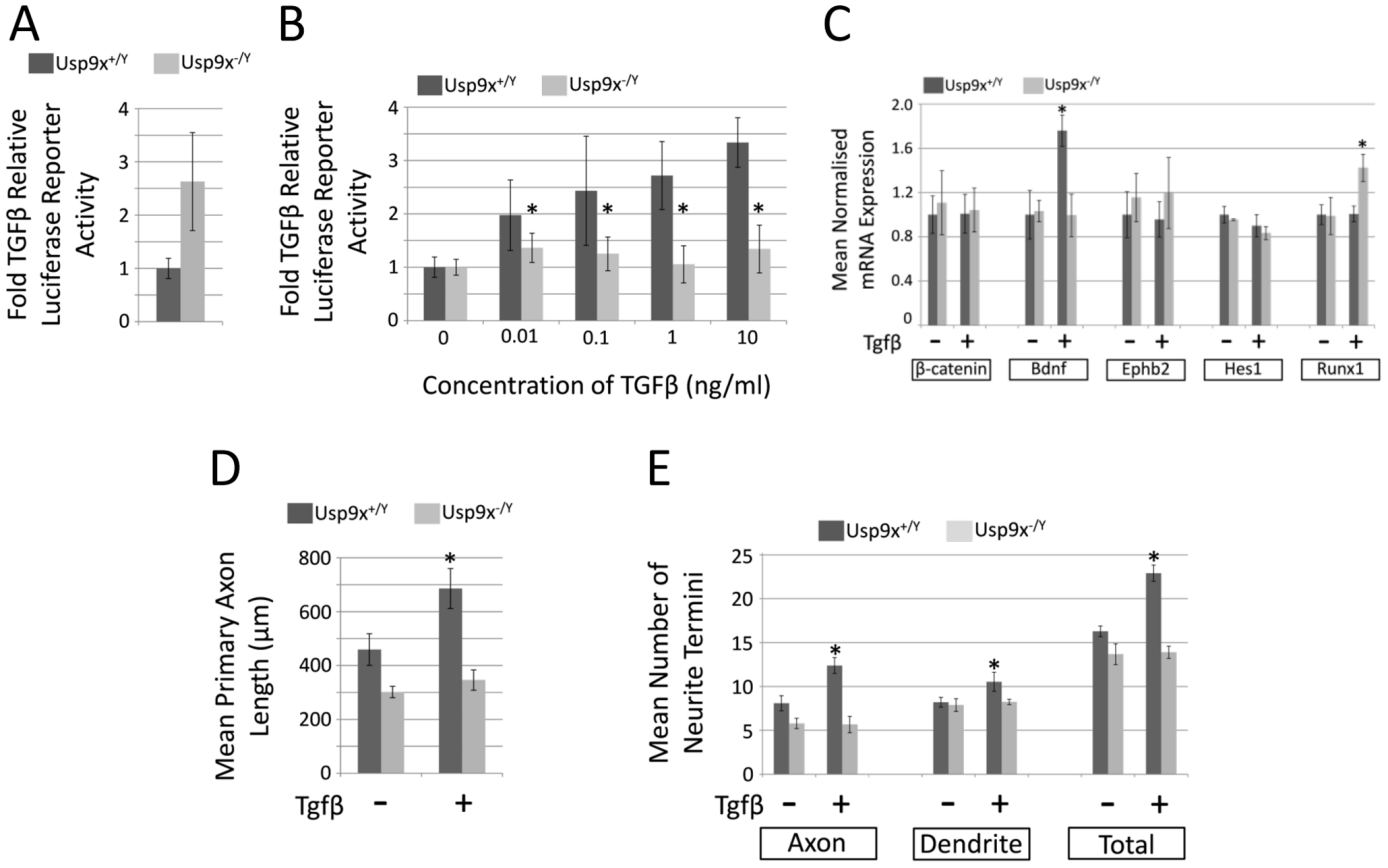
Loss of *Usp9x* disrupts TGF-β signalling in hippocampal neurons. (a–b) TGF-β luciferase reporter assays conducted in either wildtype (Nes-*Usp9x^+/Y^*) or null (Nes-*Usp9x^−/Y^*) embryonic hippocampal neuronal cultures. Hippocampal neurons were isolated and transfected with both renilla control and pGL3-TGF-β luciferase reporter plasmids. (a) Cells were grown for 3 days before analysis using dual-luciferase reporter assays and data normalised relative to wildtype readings. (b) Luciferase reporter activity in response to increasing concentrations of TGF-β. Data normalised to controls in the absence of TGF-β. All luciferase data from 6 biological replicates (i.e. cultures isolated from 6 *Usp9x^+/y^* and 6 *Usp9x^−/Y^* embryos). (c) Response of established TGFβ target genes in presence or absence of *Usp9x*, analysed by RT-qPCR. Isolated hippocampal neurons grown for 2 days prior to the addition of 1 ng/ml TGF-β. (d–e). Morphological analysis of hippocampal neurons exposed to 1 ng/ml TGF-β in the presence or absence of Usp9x. (d) Comparison of mean primary axonal length. (e). Comparison of number of neurite termini.

Usp9x is a major regulator of the Tgf-β family signalling pathway due to its deubiquitylation and hence activation of, the common Smad protein, Smad4 [27], [37]. Recently, Tgf-βr2 has been shown to be important for axon initiation and elongation [60], whilst it is well established that supplementing cultured neurons with TGFβ increases axonal growth [61]. Therefore we sought to determine if loss of Usp9x affected Tgf-β signalling in neurons (Fig. 7). To test the status of Tgf-β signalling in the absence of Usp9x we transfected hippocampal neurons with a Tgf-β-luciferase reporter construct [51]. We detected 2.6-fold higher basal levels of luciferase activity, normalised against renilla expression in the absence of Usp9x (Fig. 7A). However, while wild-type neurons could respond to increasing concentrations of Tgf-β with higher luciferase activity, there was no change in the absence of Usp9x (Fig. 7B). This difference was greatest in the presence of 10 ng/ml TGFβ, resulting in a 3.3 fold elevation of luciferase activity relative to basal levels in control neurons, compared to only a 1.3 fold elevation in *Usp9x^−/Y^* neurons. This suggested that Usp9x is required in neurons to respond to Tgf-β. We next measured the mRNA levels of putative Tgf-β target genes in response to 1 ng/ml Tgf-β in the presence and absence of Usp9x. The TGF-β target gene *Bdnf* [62] showed no response to Tgf-β in the absence of Usp9x. Bdnf acts as a self-amplifying autocrine factor to promote axon formation and growth in hippocampal neurons [63]. Paradoxically, *Runx1* showed a higher response to Tgf-β in the absence of Usp9x. However as *Runx1* responds to TGF-β in a Smad4-independent manner [64] it might not be affected by the absence of Usp9x which deubiquitytes Smad4. The other putative target genes assayed, *β-catenin*, *Ephb2* and *Hes1* did not respond to Tgf-β. Finally we measured the response of axon growth and neurite arborisation to 1 ng/ml Tgf-β exposure following 5 days of culture. Consistent with previous reports [61] we detected an 49% increase in axonal length in wild type neurons in response to Tgf-β. In comparison, only a negligible increase (14%) in axonal length was detected in Usp9x-null neurons (Fig. 7D). Likewise, whilst the number of neurite termini was increased by 40% in response to Tgf-β in control neurons, again predominately because of increases in axonal branching, no such response was observed in cells lacking Usp9x (Fig. 7C). These data establish that axon growth in hippocampal neurons requires Usp9x in order to respond to Tgf-β. Loss of Usp9x did not affect the viability of the cultured hippocampal neurons (Figure S6).

## Discussion

*Usp9x* is highly expressed throughout the embryonic brain and in specific regions of the adult brain including the neurogenic zones [16], [17]. To investigate whether *Usp9x* is required for neural development and maintenance we deleted *Usp9x* in neural progenitors of the developing brain using both a *Nestin-Cre* and *Emx1-Cre* deletion strategy. Here, we report that loss of *Usp9x* in the entire brain results in death within 24 hours of birth, possibly due to a failure to suckle. Although the overall architecture of the brain developed normally in the absence of Usp9x, its loss resulted in reduction of the corpus callosum and hippocampal size. At higher resolution, we revealed loss of Usp9x led to disorganisation of the developing neocortex architecture, observed early within the neural progenitor populations, and in the axonal projections of the neurons of the cortical plate.

Our data show that during neuronal development *Usp9x* is required for the correct development of CNS axons. The severe reduction of the corpus callosum in Emx1-*Usp9x^−/Y^* mice is consistent with this observation. To examine the molecular mechanisms underlying the axonal defect we assessed Tgf-β signalling capacity in cultured hippocampal neurons. The rationale for this was based on two observations. First, Usp9x regulation of Tgf-β signalling has been demonstrated in other developmental systems, as diverse as *Drosophila* wing development and dorsal-ventral patterning, the gastrulating *Xenopus* embryo, and wound healing in human cells [27], [37]. Usp9x regulation of these systems is due to its deubiquitylation of the common Smad protein, Smad4, which partners with receptor Smads to activate Tgf-β family member target genes [65]. Cyclic ubiquitylation and deubiquitylation of Smad4 by the E3 ligase Ectodermin and Usp9x, respectively, regulates the nuclear/cytoplasmic shuttling of Smad4 which is required for continued Tgf-β signalling in the presence of ligand [27]. The second observation is that the Tgf-βrII receptor is required for axonogenesis in the mouse brain [60]. The axonal phenotype observed in these mice is similar to those in our *Usp9x* null mice. Our data show that axons from hippocampal neurons did not increase in length in response to exogenous Tgf-β in the absence of Usp9x. In addition, a Tgf-β reporter construct and the endogenous target gene *Bdnf*, did not respond to increasing TGF-β concentration in the absence of Usp9x (Fig. 7). These data strongly suggest there is little Tgf-β signalling in neurons in the absence of Usp9x explaining, at least in part, the failure or delay in axon growth in *Usp9x* deleted brains. However, Usp9x facilitation of Tgf-β signalling does not entirely explain our observed phenotype. Deletion of *Smad4* in the brain results in a much milder phenotype than in the *Usp9x^−/Y^* mice [66]. In addition, although Tgf-βrII is required for axon development it had no effect on dendrites, but dendritic development was also impaired in the absence of Usp9x, at least *in vitro* (Fig. 4) suggesting Usp9x regulates other critical substrates during CNS development.

In addition to its requirement during axonal development, loss of Usp9x also affected the localisation of the axonal protein NF160 in the forebrain of adult *Emx1-Usp9x^−/Y^* mice. In a number of neurons NF160 co-localised with the dendritic marker MAP2 in thick processes projecting toward the pial surface (Fig. 4). This may reflect inappropriate trafficking of NF160 to dendrites, and Usp9x has been implicated in the trafficking of proteins in a polarised manner [33], [67]. Alternatively, the mis-localisation of NF160 may reflect a more general loss of polarity in Usp9x null neurons. The earliest consequence of Usp9x loss was disorganisation of neural progenitors in the VZ/SVZ of the neocortex observed in Nissl-stained sections and the tangled organisation of nestin and BLBP (Fig. 1). Usp9x regulates a number of cell adhesion and polarity proteins in polarised epithelial cells and increased expression of *Usp9x* enhances the polarised organisation of neural progenitors *in vitro* [17]. We have identified a number of cell adhesion and polarity complex proteins mis-localised or mis-expressed in *Usp9x* null neural progenitors (SP, SAW in preparation). These early effects may have ramifications for later developmental events such as axon elongation.

Loss of Usp9x also reduced the size of the hippocampus by 75% (Fig. 5). Hippocampal reduction was observed at late embryonic stages though not as severe as in the adult. An increase in apoptosis was observed in E18.5 and P0 *Emx1-Usp9x^−/Y^* in the hippocampal region and may, in part, contribute to the reduction. A potential candidate mediating this effect is the anti-apoptopic protein Mcl1, which can be stabilised by Usp9x, at least in lymphoma and cultured cell lines [68]. However, deletion of *Mcl1* results in a more severe loss of neural cells throughout the entire CNS. Therefore Usp9x may only be a rate-limiting factor of Mcl1 levels in the hippocampus. The hippocampal phenotype, in particular the reduction in size, disorganisation of the CA3 region, together with the absence or reduction of the corpus callosum, is similar to that observed in Doublecortin (*Dcx*) and Doubelcortin-like (*Dckl*) double knock-out mice [69]. Dcx is an Usp9x interacting protein, though not a substrate, as it is not ubiquitylated [16]. Instead it is proposed that Dcx traffics Usp9x along axonal microtubules. A mutation in Dcx, which is unable to bind specifically to Usp9x, was detected in a patient with lissencephaly, suggesting this interaction is important in human cortical development [16]. Given the similarity in phenotype in the mouse knockout models, it may be that Usp9x is also required for the migration of hippocampal neurons, especially those destined for the CA3 region. The disorganisation of nascent neurons in the cortical plate in embryonic cerebral cortex (Fig. 1) also supports a role for Usp9x in neuronal migration. However, this may be independent of Dcx as its expression was not altered in the cerebral cortex in the absence of Usp9x (Figure S3).

The Bmp and Wnt signalling pathways at the cortical hem in the medial neocortex are essential for the development of the hippocampus [70], [71]. As a member of the Tgf-β family of signalling proteins, Smad4 is required in the signal-receiving cell to facilitate Bmp signalling. As noted above Usp9x neurons did not respond to Tgf-β raising the possibility that defective Bmp signalling may contribute to the reduction in hippocampal growth as has been suggested [72]. Usp9x can also stabilise β-catenin, which is a second messenger for the canonical Wnt signalling pathway. Therefore Wnt signalling may also be defective in the hippocampal anlage. A more detailed analysis of Usp9x regulation of hippocampal development is required.

*USP9X* has already been linked to a number of human neurodevelopmental and neurodegenerative disorders. USP9X is linked to lissencephaly, via its interaction with DCX [16], and *USP9X* is a candidate gene in X-linked intellectual disability and epilepsy [22]. Usp9x also deubiquitylates mono-ubiquitylated alpha-synuclein raising the possibility it may play a role in the progression of neurodegenerative diseases such as Parkinson’s disease [73]. This study indicates that Usp9x regulation of brain development likely involves multiple pathways and elucidation of these mechanisms may shed light on a number of human conditions involving aberrant neural development and/or function.

## Supporting Information

Figure S1 **Deletion of Usp9x exon 3 and protein in Nes-Usp9x−/Y embryos.** (A) PCR detected removal of exon 3 in genomic DNA isolated from Nes-*Usp9x^−/Y^* E18.5 embryos. (B) Immunoblot analysis of whole brain lysate revealing decreased levels of Usp9x protein in E18.5 Nes-*Usp9x^−/Y^* embryos identified by PCR. Residual levels of full length Usp9x probably reflect the presence of non-neural cells in brain lystates. (C–F) Usp9x antibody staining of neocortex of E12.5 (C,D) and E14.5 (E,F) wild-type (Nes-*Usp9x^+/Y^*; C,E) and knockout (Nes-*Usp9x^−/Y^*; D,F) embryos. Residual amounts of Usp9x was detected in E12.5 Nes-*Usp9x^−/Y^* neural tissue (D), but Usp9x was unable to be detected by E14.5 (F). Representative images from n = 4 for each genotype at each embryonic stage.
(TIF)

Figure S2 **Usp9y expression is not induced in the absence of Usp9x.** RT-PCR failed to detect Usp9y transcripts in P0 brains in *Usp9x^+/Y^* (WT), Nes-*Usp9x^−/Y^* (cKO) of female (F) pups. Usp9y was detected in RNA isolated from adult mouse testis (T). Beta-actin transcripts were detected in all samples.
(TIF)

Figure S3 **Loss of Usp9x does not affect Doublecortin in the developing cerebral cortex.** Immunofluorescence staining of Doublecortin (Dcx) in the presence (A,C,E,G,I) or absence (B,D,F,H,J) of Usp9x. At each stage three *Usp9x^+/Y^* and three *Usp9x^−/Y^* littermates were compared. Representative images are shown. Embryos from *Nes-Usp9x* matings (A–H) and pups (postnatal day 7, P7) from *Emx1-Usp9x* matings were analyzed. LV = lateral ventricle. Scale bar = 100 µm.
(TIF)

Figure S4 **Loss of Usp9x increases neural apoptosis.** 10 µm coronal cryosections of medial neocortex from E18.5 *Nes-Usp9x* embryos stained with antibodies to cleaved caspase 3 (red) to identify cells undergoing apoptosis. Nuclei are stained with DAPI (blue).
(TIF)

Figure S5 **Emx1-cre deletion of Usp9x.** 10 µm coronal cryosections of 7 week Emx1-*Usp9x^+/y^* (A,C) and Emx1-*Usp9x* ^−/y^ (B,D) brains stained with Usp9x antibody (red) and DAPI (blue) to detect nuclei. Usp9x is absent form the cerebral cortex (B) but present at the same level in the striatum as control littermates (C,D).
(TIF)

Figure S6 **Loss of Usp9x does not affect the apoptosis of cultured hippocampal neurons.** Wildtype (*Usp9x^+/Y^*; n = 3) or knockout (*Usp9x^−/Y^*; n = −3) hippocampal neuronal cultures were grown *in-vitro* for 8 days. A. Representative immunofluorescent images showing cells stained for the apoptotic marker activated caspase 3 (green), the neuronal marker βIII-tubulin (red) and cell nuclei counterstained with DAPI (Blue). B. The percentage of caspase3 positive (+ve) cells in wildtype and Usp9x null cultures. At least 1000 cells were scored per experiment. p = 0.39 by Students 2-tailed unpaired t-test.
(TIF)
