# Supplementary figures and images for: Loss of Usp9x Disrupts Cortical Architecture, Hippocampal Development and TGFβ-Mediated Axonogenesis
Source: PLoS One. 2013 Jul 5;8(7):e68287. doi: 10.1371/journal.pone.0068287 (PMC3702552; doi:10.1371/journal.pone.0068287)

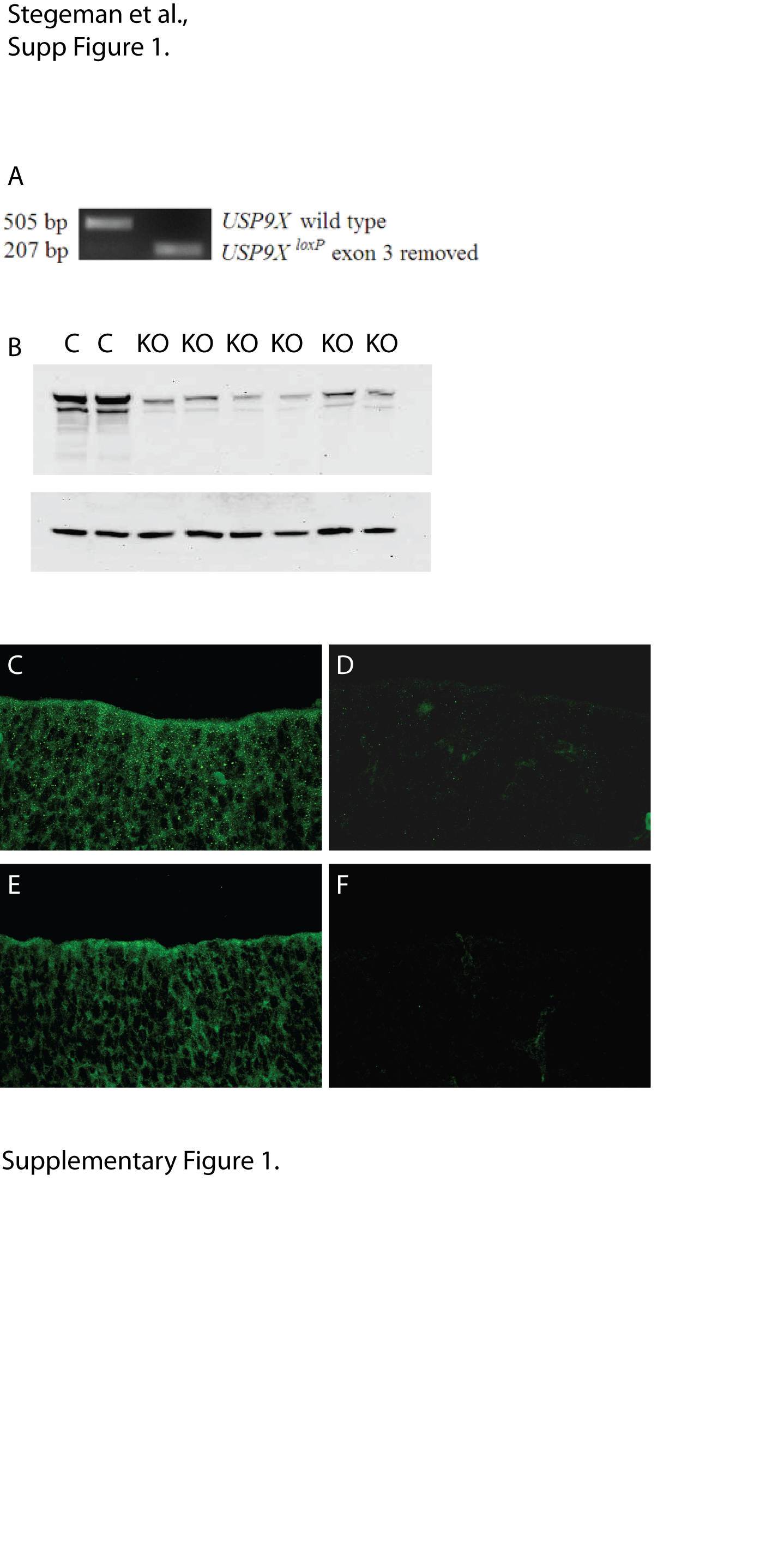

Supplement: Figure S1 — Deletion of Usp9x exon 3 and protein in Nes-Usp9x−/Y embryos. (A) PCR detected removal of exon 3 in genomic DNA isolated from Nes-Usp9x−/Y E18.5 embryos. (B) Immunoblot analysis of whole brain lysate revealing decreased levels of Usp9x protein in E18.5 Nes-Usp9x−/Y embryos identified by PCR. Residual levels of full length Usp9x probably reflect the presence of non-neural cells in brain lystates. (C–F) Usp9x antibody staining of neocortex of E12.5 (C,D) and E14.5 (E,F) wild-type (Nes-Usp9x+/Y; C,E) and knockout (Nes-Usp9x−/Y; D,F) embryos. Residual amounts of Usp9x was detected in E12.5 Nes-Usp9x−/Y neural tissue (D), but Usp9x was unable to be detected by E14.5 (F). Representative images from n = 4 for each genotype at each embryonic stage. (TIF) [file pone.0068287.s001.tif]

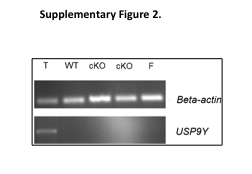

Supplement: Figure S2 — Usp9y expression is not induced in the absence of Usp9x. RT-PCR failed to detect Usp9y transcripts in P0 brains in Usp9x+/Y (WT), Nes-Usp9x−/Y (cKO) of female (F) pups. Usp9y was detected in RNA isolated from adult mouse testis (T). Beta-actin transcripts were detected in all samples. (TIF) [file pone.0068287.s002.tif]

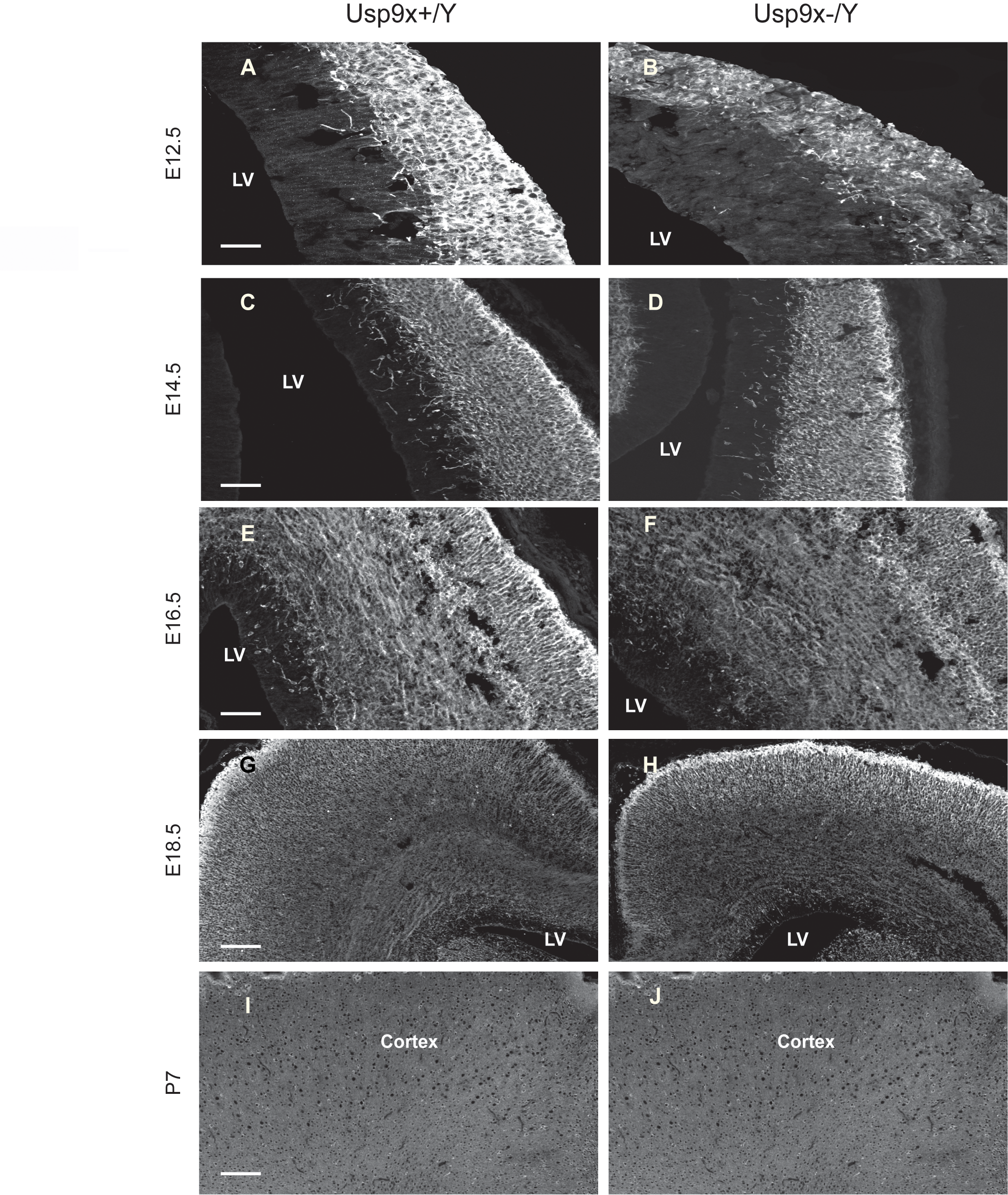

Supplement: Figure S3 — Loss of Usp9x does not affect Doublecortin in the developing cerebral cortex. Immunofluorescence staining of Doublecortin (Dcx) in the presence (A,C,E,G,I) or absence (B,D,F,H,J) of Usp9x. At each stage three Usp9x+/Y and three Usp9x−/Y littermates were compared. Representative images are shown. Embryos from Nes-Usp9x matings (A–H) and pups (postnatal day 7, P7) from Emx1-Usp9x matings were analyzed. LV = lateral ventricle. Scale bar = 100 µm. (TIF) [file pone.0068287.s003.tif]

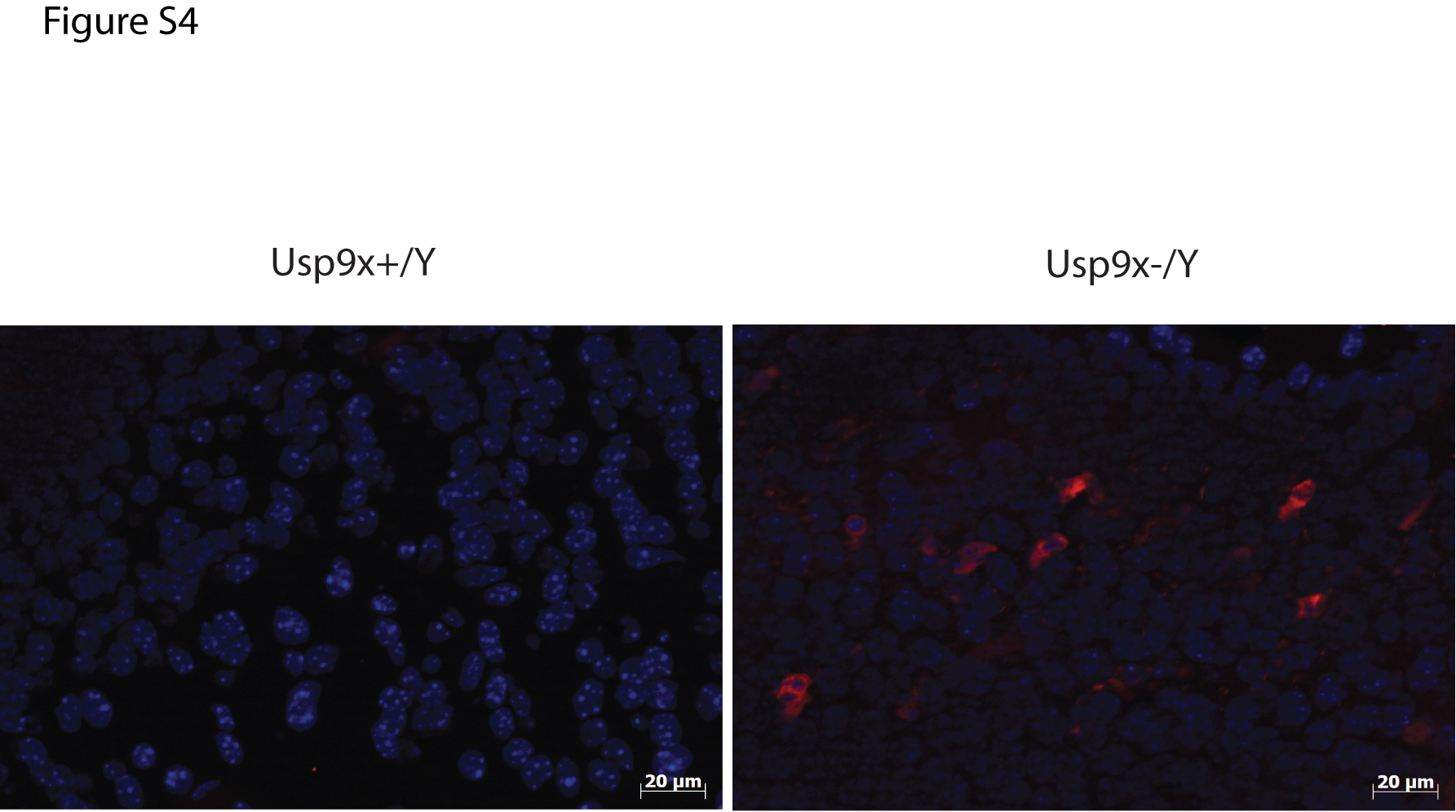

Supplement: Figure S4 — Loss of Usp9x increases neural apoptosis. 10 µm coronal cryosections of medial neocortex from E18.5 Nes-Usp9x embryos stained with antibodies to cleaved caspase 3 (red) to identify cells undergoing apoptosis. Nuclei are stained with DAPI (blue). (TIF) [file pone.0068287.s004.tif]

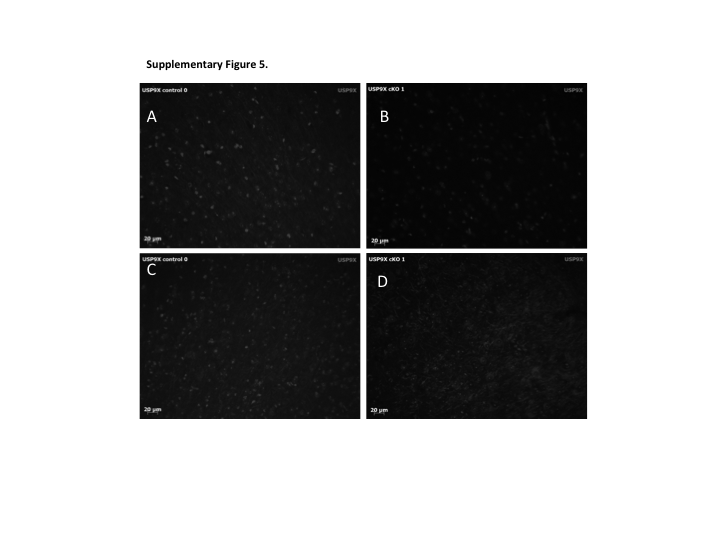

Supplement: Figure S5 — Emx1-cre deletion of Usp9x. 10 µm coronal cryosections of 7 week Emx1-Usp9x+/y (A,C) and Emx1-Usp9x −/y (B,D) brains stained with Usp9x antibody (red) and DAPI (blue) to detect nuclei. Usp9x is absent form the cerebral cortex (B) but present at the same level in the striatum as control littermates (C,D). (TIF) [file pone.0068287.s005.tif]

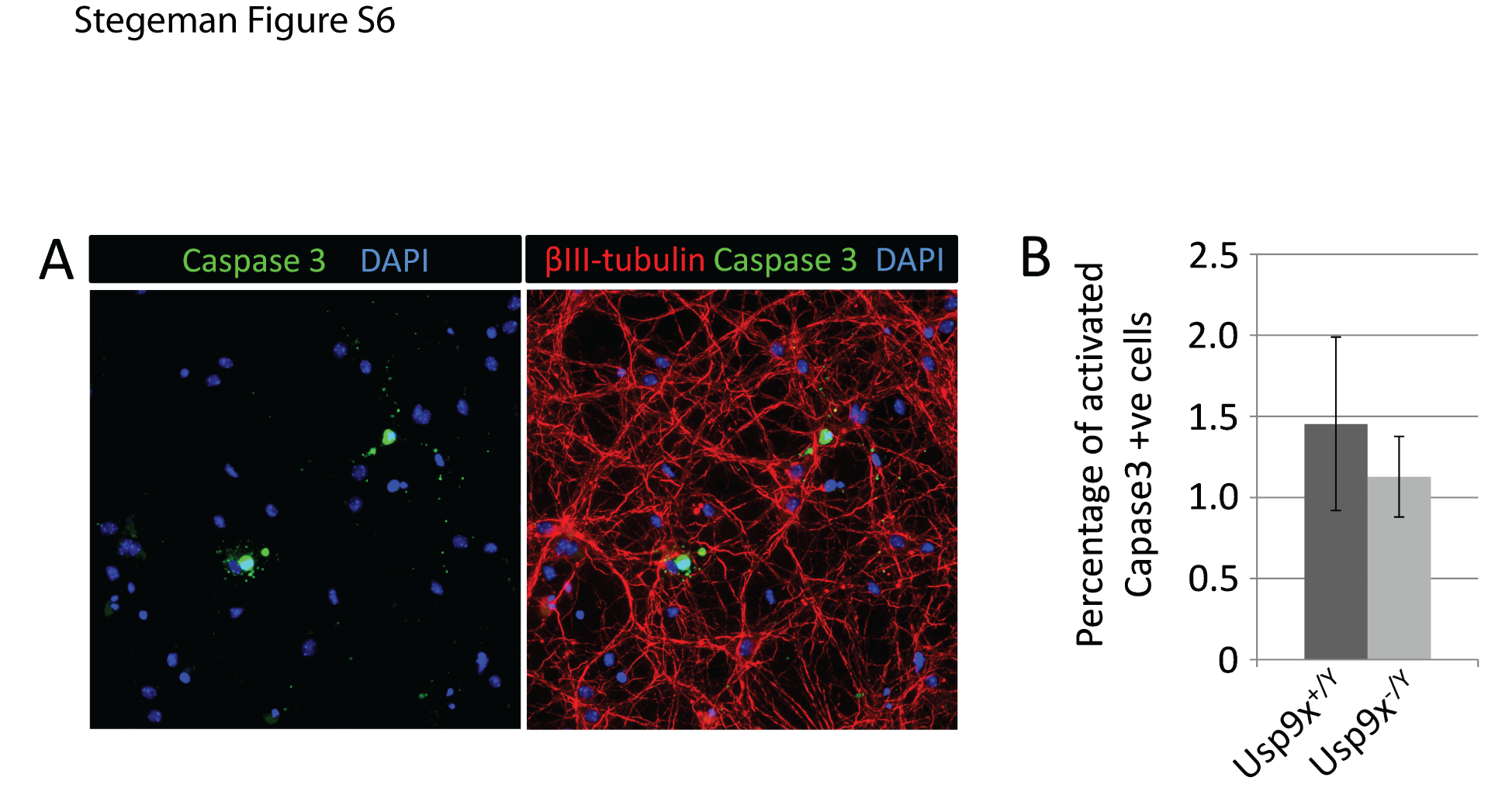

Supplement: Figure S6 — Loss of Usp9x does not affect the apoptosis of cultured hippocampal neurons. Wildtype (Usp9x+/Y; n = 3) or knockout (Usp9x−/Y; n = −3) hippocampal neuronal cultures were grown in-vitro for 8 days. A. Representative immunofluorescent images showing cells stained for the apoptotic marker activated caspase 3 (green), the neuronal marker βIII-tubulin (red) and cell nuclei counterstained with DAPI (Blue). B. The percentage of caspase3 positive (+ve) cells in wildtype and Usp9x null cultures. At least 1000 cells were scored per experiment. p = 0.39 by Students 2-tailed unpaired t-test. (TIF) [file pone.0068287.s006.tif]
